# Magneto-Ionics in Single-Layer Transition Metal Nitrides

**DOI:** 10.1021/acsami.1c06138

**Published:** 2021-06-22

**Authors:** Julius de Rojas, Joaquín Salguero, Fatima Ibrahim, Mairbek Chshiev, Alberto Quintana, Aitor Lopeandia, Maciej O. Liedke, Maik Butterling, Eric Hirschmann, Andreas Wagner, Llibertat Abad, José L. Costa-Krämer, Enric Menéndez, Jordi Sort

**Affiliations:** † Departament de Física, Universitat Autònoma de Barcelona, Cerdanyola del Vallès E-08193, Spain; ‡ IMN-Instituto de Micro y Nanotecnología (CNM-CSIC), Isaac Newton 8, PTM, Tres Cantos, Madrid 28760, Spain; § Univwesity of Grenoble Alpes, CEA, CNRS, Spintec, Grenoble 38000, France; ∥ Institut Universitaire de France, Paris 75231, France; ⊥ Department of Physics, Georgetown University, Washington, District of Columbia 20057, United States; # Catalan Institute of Nanoscience and Nanotechnology (ICN2), Campus UAB, Bellaterra, Barcelona E-08193, Spain; ∇ Institute of Radiation Physics, Helmholtz-Zentrum Dresden−Rossendorf, Dresden 01328, Germany; ◊ Institut de Microelectrònica de Barcelona, IMB-CNM (CSIC), Campus UAB, Bellaterra, Barcelona E-08193, Spain; ○ Institució Catalana de Recerca i Estudis Avançats (ICREA), Pg. Lluís Companys 23, Barcelona E-08010, Spain

**Keywords:** voltage control of magnetism, magneto-ionics, electrolyte gating, transition metals, metal nitrides, nitrogen ions, open volume defects, positron annihilation spectroscopy

## Abstract

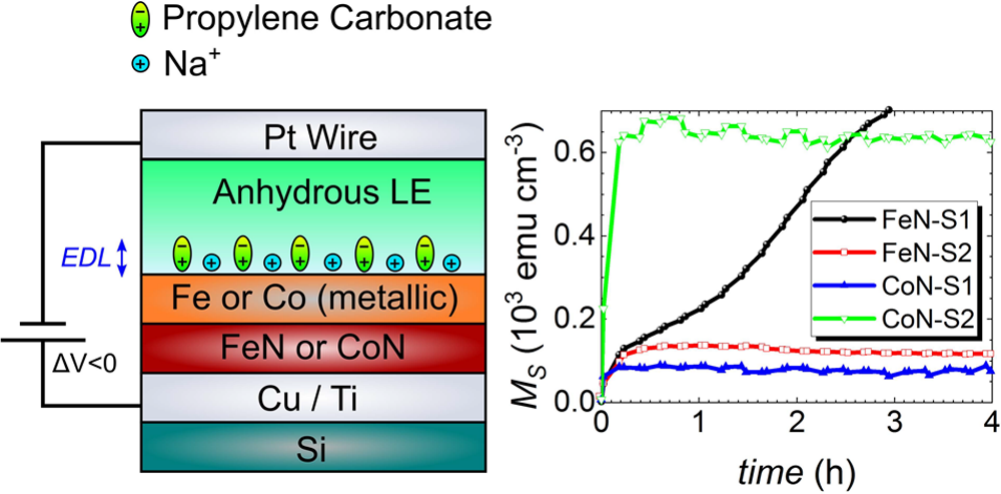

Magneto-ionics allows for tunable control of magnetism by voltage-driven transport of ions, traditionally oxygen or lithium and, more recently, hydrogen, fluorine, or nitrogen. Here, magneto-ionic effects in single-layer iron nitride films are demonstrated, and their performance is evaluated at room temperature and compared with previously studied cobalt nitrides. Iron nitrides require increased activation energy and, under high bias, exhibit more modest rates of magneto-ionic motion than cobalt nitrides. Ab initio calculations reveal that, based on the atomic bonding strength, the critical field required to induce nitrogen-ion motion is higher in iron nitrides (≈6.6 V nm^–1^) than in cobalt nitrides (≈5.3 V nm^–1^). Nonetheless, under large bias (i.e., well above the magneto-ionic onset and, thus, when magneto-ionics is fully activated), iron nitride films exhibit enhanced coercivity and larger generated saturation magnetization, surpassing many of the features of cobalt nitrides. The microstructural effects responsible for these enhanced magneto-ionic effects are discussed. These results open up the potential integration of magneto-ionics in existing nitride semiconductor materials in view of advanced memory system architectures.

## Introduction

Modern electronic devices store data using electric current to manipulate the magnetization orientation of magnetic domains. With device miniaturization pushing nominal device dimensions toward 10 nm, greater amounts of energy are expended due to resistive heating and device cooling. This challenge has spurred research toward discovering novel materials and developing new devices for next-generation technologies with improved energy efficiency, robust thermal stability, and precise control of magnetic properties. Voltage-controlled magnetism (VCM) tackles this challenge by replacing electric current with an applied voltage, potentially leading to significant energy savings.^1,2^ Magneto-ionics,^3−18^ a branch of VCM in which ions such as O^2–^, H^+^, Li^+^, F^–^, or N^2–/3–^ are injected into and withdrawn from a target material under an applied bias, has been shown to be capable of generating large, nonvolatile, and reproducible modulations of bulk magnetic observables. A typical magneto-ionic structure is composed of a ferromagnetic layer in contact with an oxide reservoir layer from which oxygen ions are transported, modifying the target material’s structure and stoichiometry, with corresponding changes in coercive field, exchange bias field, magnetic easy axis, or magnetic anisotropy.^8−10,12,17,19−23^ Cyclability remains an issue as ionic transport may result in irreversible structural changes. Recently, ionic motion on the order of 10^3^ Hz has been successfully demonstrated with good endurance via a proton-based (H^+^) mechanism, albeit with some limitations in the operational film thicknesses and hydrogen retention.^11^

Recent studies of the magneto-ionic properties of single-layer thin films with structural oxygen (Co_3_O_4_) or nitrogen (CoN) ions, present in the as-prepared thin-film state, have demonstrated that fully reversible and cyclable magnetic transitions between a nonferromagnetic (OFF) and a ferromagnetic state (ON) are indeed possible.^5,12,24^ Interestingly, and in contrast to the diffusion channels observed in Co_3_O_4_, CoN films transport nitrogen via a planar ion migration front and possess both superior cyclability and lower operating voltages than Co_3_O_4_,^24^ hinting that metal nitrides may compare favorably with their metal oxide counterparts. Previous ab initio calculations of the enthalpy of formation of CoN have predicted values of *ΔH*_f_ ≈ −50 kJ mol^–1^,^25^ significantly higher (i.e., less negative) than experimental estimates conducted on CoO and Co_3_O_4_ of *ΔH*_f_ ≈ −237.9 and −910.02 kJ mol^–1^, respectively.^26−28^ This is consistent with the difference in electronegativity between nitrogen and oxygen: the lower electronegativity of nitrogen results in weaker bonds with Co cations, suggesting increased magneto-ionic mobility. Properly tuned FeN may be a tantalizing alternative target material for magneto-ionics, as ab initio calculations show that the enthalpy of formation of FeN is comparable to that of CoN and significantly higher (i.e., less negative) than that of FeO.^25,29^ In addition, magnetic nitrides^30,31^ such as Fe–N^32−37^ have recently drawn significant research interest due to their array of desirable properties, including high hardness, melting point, incompressibility, cost efficiency, and greater magnetization than iron oxides,^38−43^ another class of magneto-ionic target materials.^18,21,23^ Iron nitrides also span a wide range of mechanical and magnetic properties, which can be tuned by varying the nitrogen concentration in Fe_*x*_N_*y*_,^44−47^ and can be easily integrated with semiconductor electronics. These factors, along with the relative abundance of Fe over Co,^48^ suggest iron nitride could be a prime candidate for magneto-ionics.

In this work, voltage-driven nitrogen transport in iron nitride films is demonstrated, and the magneto-ionic performance is evaluated and compared with cobalt nitride films. The iron nitride films are found to have, under large bias (i.e., well above the magneto-ionic onset voltage and, thus, when magneto-ionics is fully activated), greater total magnetizations, larger coercive fields, lower magneto-ionic rates, and lower (i.e., more negative) onset voltages than the examined cobalt nitrides (−8 vs −4 V). The microstructural effects responsible for the enhanced magneto-ionically induced coercivity and magnetization in iron nitride films are discussed, while ab initio calculations reveal that the formation energy of FeN requires a greater critical field (≈6.6 V nm^–1^) to induce magneto-ionic motion than CoN (≈5.3 V nm^–1^), consistent with the experimentally observed voltages needed to initiate magneto-ionics.

## Results and Discussion

Iron nitride (FeN) and cobalt nitride (CoN) films (85 nm) were grown atop Cu (60 nm)/Ti (20 nm)/[1 0 0]-oriented Si substrates. Deposition of iron and cobalt was performed using nitrogen partial pressures of 25% and 50%, respectively, resulting in differing nanocrystallinities and electrical transport behavior. To distinguish between the films by nitrogen partial pressure and relative electric resistivity, they are labeled FeN–S1, FeN–S2, Co–S1, and Co–S2 (see Table 1).

**Table 1:** Iron and Cobalt Nitride Films, Argon/Nitrogen Partial Pressure during Deposition, Crystallite Size, and Resistivity at Room Temperature^a^

Co–N	P_N2_ (%)	N concentration (%)	⟨*D*⟩ (nm)	ρ (*T* = 300 K) (μΩ·cm)
FeN–S1	25	49	10	304
FeN–S2	50	51	14	509
CoN–S1	25	33	9	156
CoN–S2	50	50	13	411

a Crystallite sizes obtained from Rietveld refinement of the X-ray diffraction patterns.^51^ For comparison, the resistivities of pure Fe and Co films are 21 and 11 μΩ·cm, respectively (measured at room temperature).

**Figure 1:**
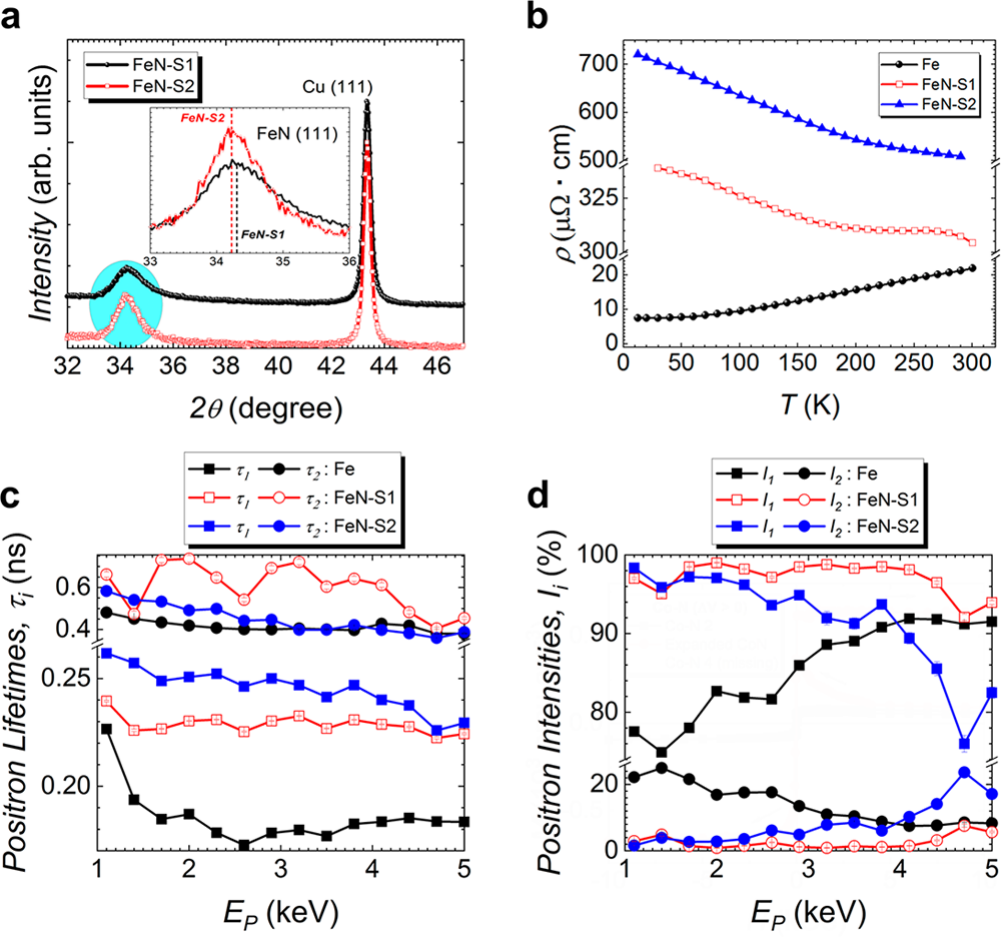
Structural, transport, and defect characterization of iron nitride films. (a) θ/2θ XRD diffraction patterns of the as-prepared iron nitride films. (b) Resistivity ρ measured as a function of temperature from 20 to 300 K for all as-prepared iron nitride films. (c and d) Positron lifetime components *τ*_*i*=1–2_ and their relative intensities *I*_*i*=1–2_ as a function of positron implantation energy *E*_*P*_ for all as-prepared iron nitride films.

Structural characterization of the films was carried out using θ/2θ X-ray diffraction (XRD). Figure 1a shows the XRD patterns of the as-prepared iron nitride films. The nearly stoichiometric FeN films (i.e., FeN–S1, FeN–S2) exhibit a single peak which is consistent with the (1 1 1) textured hexagonal close-packed (hcp) *P*6_3_/*mmc* FeN diffraction peak (Materials Project ID 12120) or possibly the (0 0 1) face-centered cubic (fcc) *F*4̅3*m* FeN diffraction peak (Materials Project ID 6988).^49^ The patterns also show the (1 1 1) Cu peak arising from the buffer layer. Rietveld refinement of the FeN XRD patterns reveals that the hcp phases of both films are distorted, and the films are highly nanocrystalline, with the smallest crystallite sizes in the range of 10–14 nm.. The nitrogen content is estimated by electron energy loss spectroscopy (EELS) (see Table 1). As reported earlier,^24^ the CoN–S2 film exhibits a single peak which is consistent with the (1 1 1) diffraction peak of the expanded *Fm*3*m* cubic CoN phase (see Figure S1), while CoN–S1 shows a peak consistent with hexagonal (1 0 1) Co_3_N_1+*x*_ phase.^50^ Crystallite size and nitrogen concentration values are listed for comparison (Table 1).

Resistivity measurements as a function of temperature (20–300 K) were performed on both as-prepared FeN films as well as purely metallic iron for reference (Figure 1b). The resistivity ρ at room temperature ranges from ≈21 μΩ·cm in pure Fe to ≈500 μΩ·cm in FeN–S2, increasing gradually with N content, as expected. The pure iron film shows a monotonic increase of resistivity throughout the temperature range (d*ρ*/d*T* > 0, where ρ and *T* are resistivity and temperature, respectively), consistent with metallic behavior. In the case of FeN–S2 (Figure 1b), resistivity is observed to monotonically decrease (d*ρ*/d*T* < 0) throughout the temperature range, consistent with semiconducting behavior. In contrast with FeN–S2, FeN–S1 has an overall lower resistivity than FeN–S2 with the sign of d*ρ*/d*T* negative up to 220 K, then basically zero up to 270 K, and finally negative to 300 K. This exhibits a semiconducting contribution and thus a more complex transport behavior than a typical insulating film. These relative differences in behavior mark the FeN–S1 film as relatively conductive and the FeN–S2 film as relatively resistive. CoN–S2 exhibits a similar transition in electrical transport behavior (see Table 1, Figure S2), whereas CoN–S1 is rather conductive, although with a higher electric resistivity than metallic Co.

In order to characterize the depth-dependent defect structure of the as-prepared iron nitride films, variable energy positron annihilation lifetime spectroscopy (VEPALS) experiments were conducted.^52−57^ Contributions from positron annihilation lifetimes^12^*τ*_1_, corresponding to localized vacancies, and *τ*_*2*_, corresponding to a mixture of signals from surface states and grain boundaries, are seen in both films (Figure 1c and 1d). The annihilation lifetimes, *τ*_*i*_, decrease as the positrons penetrate deeper into the film, ascribed to an increase in vacancy size close to the electrolyte-side surface of the FeN films and a decrease in vacancy size deeper in the films, rendering a graded defect structure. The positron lifetimes, *τ*_1_ and *τ*_2_, increase with resistivity, indicating that resistivity is tightly related to vacancy and grain boundary formation (i.e., the nanostructuring of the films). Examining the as-prepared FeN–S1 film, only contributions from *τ*_1_ and *τ*_2_ are observed, with no contributions from lifetime *τ*_3_ (“void-like” structures) present.^12^*τ*_1_, representing small vacancy clusters, reaches a maximum of ≈0.24 ns near the surface, before dropping to ≈0.23 ns in the film region (Figure 1c, open squares). This suggests a higher density larger defect complex size consisting of 3–4 vacancies within a cluster near the top of the film and 2–3 vacancies within a cluster near the working electrode (see Figure S3 for details). *τ*_*2*_, representing a convolution of surface states (subsurface region) and grain boundaries, remains above 0.4 ns throughout the film, indicating the presence of larger vacancy clusters and small voids near the surface. FeN–S1 shows a residual contribution from larger vacancy complexes, whereas FeN–S2 exhibits a larger density of open volumes closer to the interface with the buffer layer. The relative intensity *I*_1_’s more rapid decrease (*I*_2_ increases) is consistent with the appearance of the increasing influence of the Cu and Ti layers as the positron implantation energy approaches *E*_p_ = 4 keV (Figure 1d). This overall behavior of both of the as-prepared iron nitride films in the VEPALS measurements shows that FeN–S1 and FeN–S2 are comparable to the as-prepared state of CoN–S2 (Figure S4) with slightly lower lifetimes.

**Figure 2:**
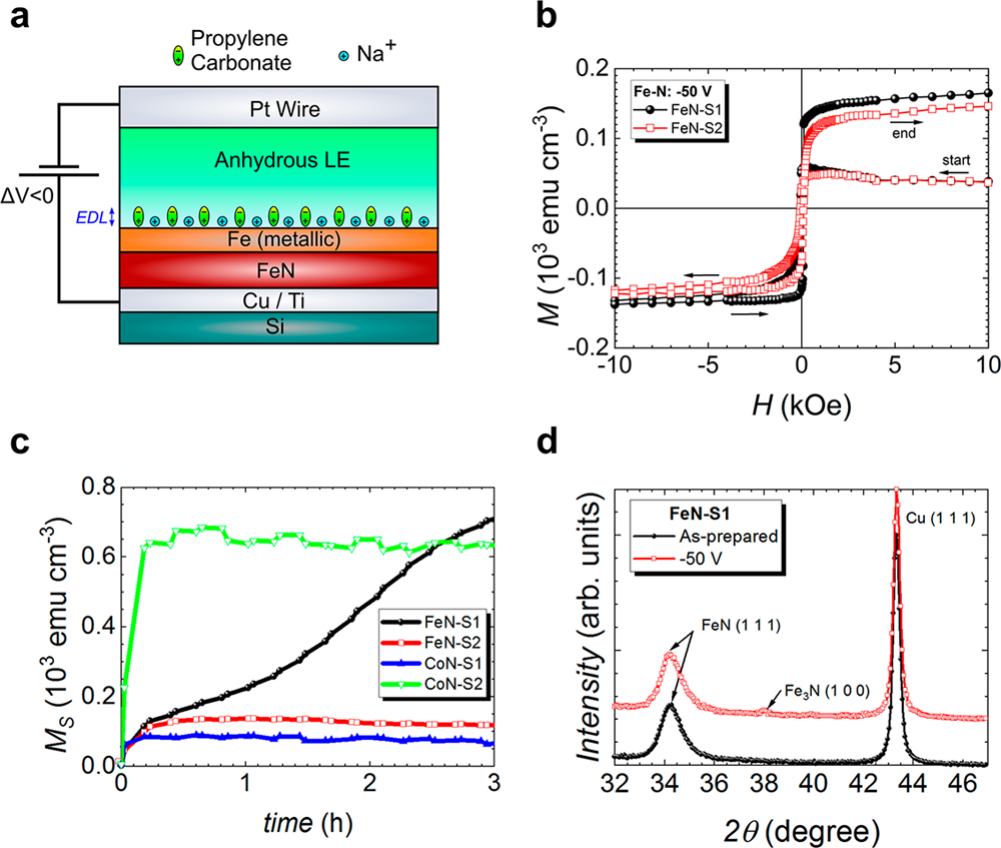
Magneto-ionic characterization of iron nitride films by in-plane vibrating sample magnetometry under electrolyte gating. (a) Schematic of the capacitor-like structure used during the measurement of each film. Anhydrous liquid electrolyte (LE) forms an electric double layer (EDL) at the film surface. Thin layer of metallic iron is drawn to represent the appearance of a ferromagnetic layer after voltage actuation. (b) First hysteresis loops under −50 V bias for iron nitride films. (c) Saturation magnetization (*M*_S_) measured as a function of time for iron nitride and cobalt nitride films, taken from *M–H* loops measured between −20 and 20 kOe. (d) θ/2θ XRD diffraction patterns of FeN–S1 before and after −50 V biasing treatment for 75 min.

All magneto-ionic measurements were preformed using a liquid electrolyte (i.e., propylene carbonate with Na^+^) in a capacitor-like configuration (Figure 2a), where the Cu/Ti buffer layer acts as a working electrode and the Pt wire acts as a counter electrode. The use of a nonaqueous, aprotic polar liquid electrolyte will prevent electrochemical oxidation during voltage biasing.^5,10,12,24^ This configuration generates an electric double layer (EDL) at the film surface which applies a uniform, out-of-plane electric field.^5,10,58−62^ In-plane measurements of *M–H* hysteresis loops, spanning the range between −20 and 20 kOe, were measured using a vibrating sample magnetometer. The upper and lower branches were measured in 12.5 min for a total of 25 min for each major loop. As-prepared FeN–S1, Fe–S2, CoN–S1, and CoN–S2 films show residual ferromagnetic behavior and a saturation magnetization *M*_*S*_ between 3 and 12 emu cm^–3^ corresponding to contamination of <0.70% (0.42%) by volume of metallic iron (cobalt), a result of off-stoichiometric regions in the film and/or ferromagnetic impurities in the substrate (Figures S5a and S5b). Magneto-ionic rates were assessed by measuring magnetic hysteresis loops as a voltage of −50 V was applied across the film.

The first *M–H* hysteresis loop measured for each iron nitride film is shown in Figure 2b. The films clearly demonstrate the appearance of a ferromagnetic phase with an increase in *M*_S_ and the coercive field *H*_C_. During the first loop the magnetization of both FeN–S1 and FeN–S2 clearly increases, although more in FeN–S1 than in FeN–S2 (Figure 2b).

The first two loops measured during −50 V biasing are plotted in Figure S5c–f, so that the changes in magnetic behavior can be more clearly observed for all films. In Figure 2c, the overall change in *M*_S_ is plotted as a function of time for FeN–S1, FeN–S2, CoN–S1, and CoN–S2. Cobalt nitride films are included for ease of comparison. For CoN–S2 and FeN–S2 films, *M*_S_ quickly increases during the initial stages of voltage application (first 12 min, Figure 2c) and subsequently levels off. Interestingly, FeN–S1 exhibits a distinct multistep process: in the first stage, *M*_S_ increases at a rate on the order of FeN–S2, which can be ascribed to the formation of a ferromagnetic Fe_3_N phase (Figure 2d), before continuing to increase, albeit at a slower rate, up to a maximum *M*_S_ (898 emu cm^–3^). The *M*_S_ values reached by each film after long-term biasing (6 h) are presented in Table 2. The initial slope of the magnetization was fitted using a linear regression with rates found to be 502 emu cm^–3^ h^–1^ for FeN–S1 and 267 emu cm^–3^ h^–1^ for FeN–S2, evidencing magneto-ionics in FeN, although with lower rates than CoN–S1 and CoN–S2 (Table 2). CoN–S2 shows a tremendous increase in *M*_S_ while sweeping through the first quadrant of the hysteresis loop under bias, greater than FeN–S1 and FeN–S2. Both cobalt nitride films show clear increases in *M*_S_ as a function of time with rates of 1012 emu cm^–3^ h^–1^ for CoN–S1 and 2602 emu cm^–3^ h^–1^ for CoN–S2 and CoN–S1 and CoN–S2 reaching final values of *M*_S_ near 90 and 637 emu cm^–3^, respectively.

**Table 2:** Magneto-Ionic Rates, Saturation Magnetization, Squareness (Ratio of Remanence to Saturation Magnetization, *M*_R_*/M*_S_), Normalized Slope at the Coercive Field, and Coercive Field of Treated Iron and Cobalt Nitride Films^a^

Co–N	d*M*/d*t* (emu cm^–3^ h^–1^)	*M*_S_ (emu cm^–3^)	*M*_R_/*M*_S_ (%)	1/*M*_S_ d*M*/d*H*@HC (kOe^–1^)	*H*_C_ (Oe)
FeN–S1	502	898	87	17	86
FeN–S2	267	137	63	5	140
CoN–S1	1012	90	84	21	65
CoN–S2	2602	637	96	97	17

a Values listed reached after 6 h of gating under −50 V bias.

The lower magneto-ionic rates of iron nitrides can be attributed to a convolution of several factors: a slightly lower enthalpy of formation of FeN, requiring greater energy to break the bonds between iron and nitrogen,^25,63^ reduced positron lifetimes *τ*_1_ and *τ*_2_, which can be correlated with smaller vacancy clusters and grain boundaries and thus a reduced ionic mobility, and variation in electrical transport properties. An increase in the required energy to separate Fe and N in turn requires a greater applied electric field (voltage) to the film, reducing the ionic drift velocity at a given voltage when compared with cobalt nitride. Smaller vacancies and grain boundaries (cross sections) reduce the conductivity of the ionic pathways, requiring greater voltage to transfer ions through the film, again reducing the drift velocity at a given voltage when compared with cobalt nitride. A higher resistivity allows the applied electric field to penetrate deeper into the film, which most likely boosts magneto-ionic rates. Higher resistivity can also be correlated with increased bonding between Fe and N, thereby increasing the energy required to begin ionic motion and thus reducing magneto-ionic rates. A balance between these factors must be achieved to optimize magneto-ionic performance. Since FeN–S1 shows a greater magneto-ionic rate than FeN–S2, XRD diffraction was carried on the FeN–S1 film post-treatment (Figure 2d). After biasing at −50 V for 75 min, FeN–S1 shows traces of a new peak compatible with an iron nitride with lower nitrogen concentration: (1 0 0) Fe_3_N (PDF 00-001-1236) or (1 0 0) Fe_2_N (PDF 00-002-1206). Indeed, the increase of magnetization *M*_S_ of FeN–S1 as nitrogen is removed from the film can be correlated with the appearance of a magnetic Fe_3_N phase, whose magnetic moment has been calculated ab initio to be on the order of 1.44 μ_B_.^64^ After long-term voltage application, the *M*_S_ of FeN–S1 is found to be larger than the *M*_S_ of the Co nitrides. Considering that the ratios of Fe:N and Co:N are nearly identical, this can be attributed to the larger magnetic moment per atom of metallic Fe (2.22 μ_B_) compared to metallic Co (1.72 μ_B_),^48^ which becomes increasingly present as more and more nitrogen is removed from the system.

The evolution of the shape of the *M*–*H* loops under bias can also provide key insights into the differences between the generated ferromagnetic phases. The squareness, defined as the ratio of the remanence, *M*_R_, to *M*_S_ (*M*_R_/*M*_S_), the slope of the hysteresis loop at the coercive field, *H*_C_, normalized to *M*_S_ (*M*_S_^–1^d*M*/d*H* [*H* = *H*_C_]), and *H*_C_ have been assessed for each branch of the measured *M–H* loops as a function of time (Figure S6a–f). The asymptotic values of the magnetic parameters reached by the iron and cobalt nitride films after 6 h of biasing at −50 V are reported in Table 2. Broadly, greater squareness and lower coercive fields indicate a steeper slope of the *M*–*H* curve and a narrower, more uniform coercive field distribution.^65^ FeN–S1 demonstrates lower squareness and a higher coercive field than CoN–S2 (Figure S6), correlating with a less uniform coercive field distribution than CoN–S2. The coercive fields of each film also reflect intrinsic differences in the generation of magnetic states. The coercive fields of FeN–S2 and CoN–S1 monotonically reach the highest values of the iron and cobalt films, respectively (140 and 65 Oe). This can perhaps be attributed to a paramagnetic to ferromagnetic transition or possibly the formation of a high density of small, isolated clusters developing from the superparamagnetic state of small, weakly coupled grains in a nonmagnetic nitride matrix. The behavior of FeN–S1 and CoN–S2 (Figure S6) resembles the well-known variation of coercivity with increasing size of a single particle, first increasing from a superparamagnetic to a single domain state and then decreasing *H*_C_(65) as the multidomain state is reached. FeN–S1 and CoN–S2 differ, however, in the maximum coercive field and time scales: FeN–S1 reaches a larger coercive field *H*_C_ more slowly (148 Oe in 2 h vs 50 Oe in 12 min in CoN–S2). Both films asymptotically approach a lower coercivity with increasing biasing time. It should be noted that FeN–S1 reaches a higher coercive peak than the coercive peaks of CoN–S1 and CoN–S2, suggesting that FeN–S1 may be more useful for magnetic memory applications, which often require magnetic stability. In general, both FeN films reached higher *H*_C_ values than either CoN film throughout the biasing process. This can be indicative of comparatively smaller activated magnetic regions, comprising single- or few-domain structures in mostly denitrided regions, perhaps the result of a less complete, planar ionic diffusion front in the iron nitride films.

**Figure 3:**
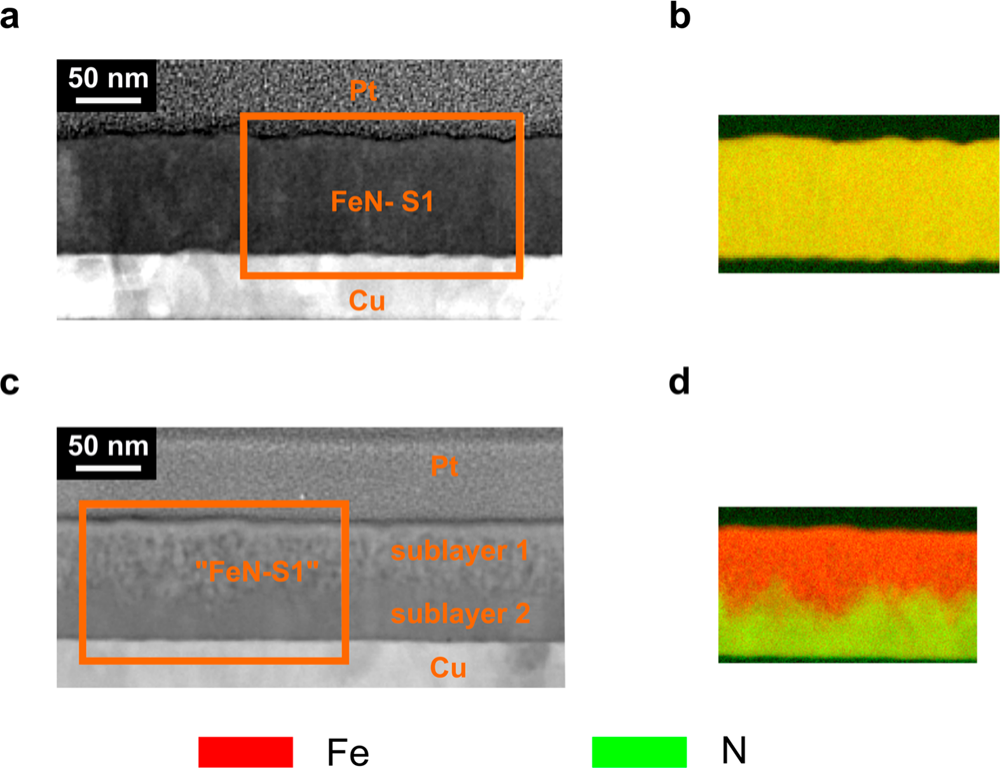
Structural and compositional characterization via high-angle annular dark-field scanning transmission electron microscopy (HAADF-STEM, left images) and electron energy loss spectroscopy (EELS, right images). HAADF-STEM and elemental EELS mappings corresponding to the areas marked in orange in as-prepared (a and b) FeN–S1and (c and d) FeN–S1 films negatively biased at −50 V for 75 min, respectively. Copper and platinum layers serve as the working electrode and a protective capping layer for lamellae preparation, respectively. Colors corresponding to each element are noted at the bottom of the figure.

To further understand the structural and compositional changes the iron nitride films undergo during gating, cross-sectional lamellae of the as-prepared and treated FeN–S1 films, capped with a protective platinum layer, were characterized by high-angle annular dark-field scanning transmission electron microscopy (HAADF-STEM) and EELS. Images of FeN–S1 are shown in Figure 3; analogous images of CoN–S2 can be found in a previous work.^24^ The as-prepared FeN–S1 film is highly nanostructured and isotropic (Figure 3a). Fe (red) and N (green) are homogeneously distributed in the films (Figure 3b), similar to the CoN–S2 as-deposited films. After treatment with −50 V for 75 min, FeN–S1 undergoes a moderate change, roughly corresponding to a more nanoporous structure closer to the electrolyte side and a highly nanostructured structure near the working electrode side, suggesting a moderate denitriding process (Figure 3c). A concentration front appears, reflected in the homogeneous presence of Fe and N (red and green, respectively) near the electrode and the reduced presence of N (orange/red), consistent with the formation of Fe_3_N near the electrolyte side (Figure 3d). Conversely, CoN–S2 has been shown to undergo a dramatic change with almost complete denitriding under −50 V for 75 min but again with a clearly defined concentration front appearing parallel to the surface.^24^ In FeN–S1, the migration front is less defined but relatively planar without the existence of cross-sectional channels as happens in other magneto-ionic systems such as Co_3_O_4_.^12^

**Figure 4:**
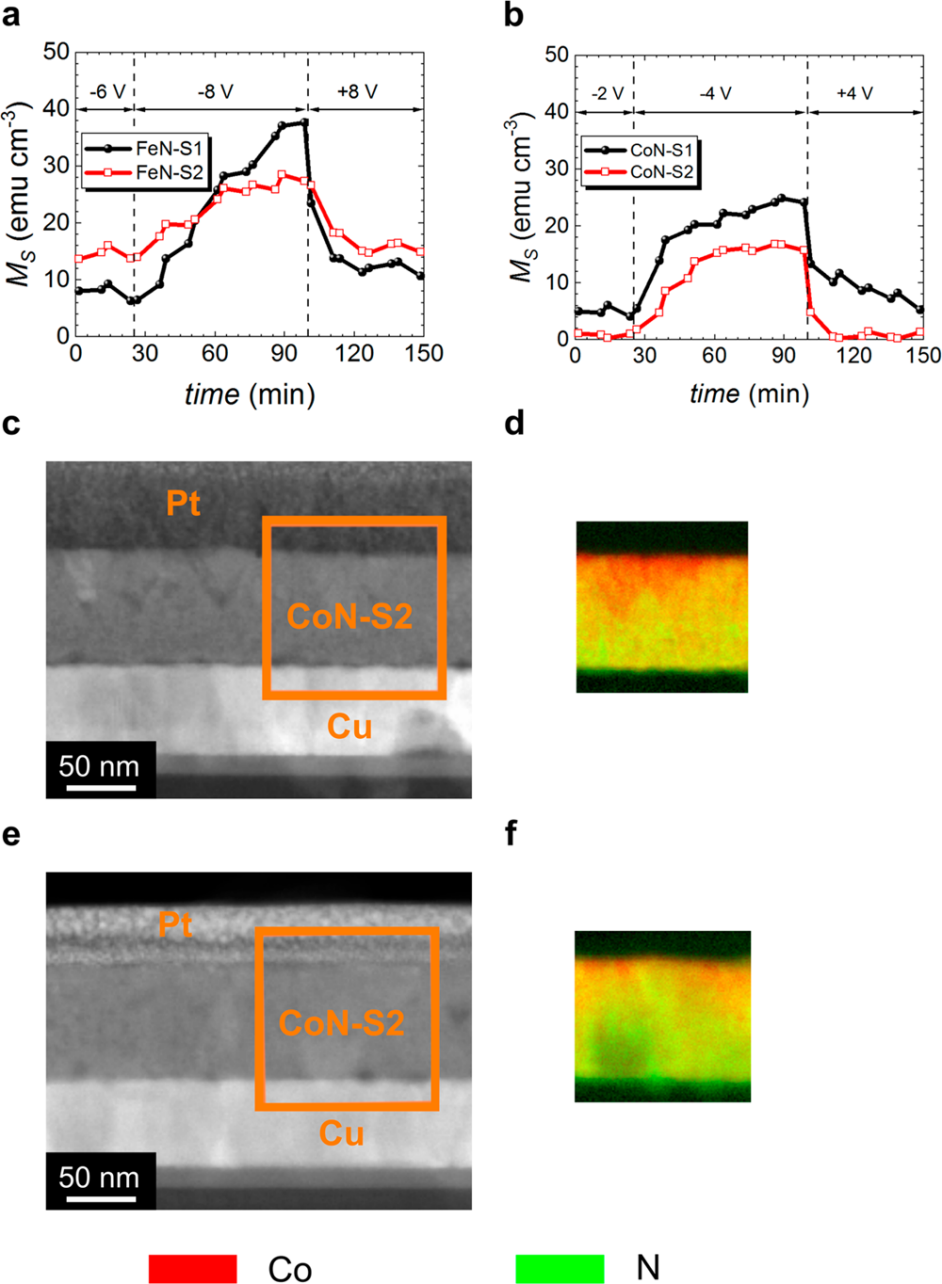
Magneto-ionic behavior of iron and cobalt nitrides during onset and recovery experiments. Structural and compositional characterization in CoN–S1 film using high-angle annular dark-field scanning transmission electron microscopy (HAADF-STEM) and electron energy loss spectroscopy (EELS). (a and b), Saturation magnetization (*M*_S_) as a function of time under applied onset and recovery voltages for FeN–S1, FeN–S2, and CoN–S1, CoN–S2. HAADF-STEM and elemental EELS mappings corresponding to the areas marked in orange in (c and d) CoN–S2 negatively biased at −4 V and (e and f) CoN–S2 subsequently positively biased at +4 V for 75 min, respectively. Colors corresponding to each element are noted at the bottom of the figure.

Understanding the minimum voltage required to initiate magneto-ionic motion is key to evaluating the magneto-ionic performance, so the onset voltages were determined by monotonically increasing the applied bias in steps of −2 V (for 25 min each) and noting when the films started to show an increase in ferromagnetic signal (Figure 4a and 4b). Both the magnitude and the duration of the applied voltage affect the magneto-ionic response, so the measured threshold voltages should be taken as approximate values. Once a threshold voltage was reached (i.e., permanent magnetism is induced), the applied voltage was held for 75 min before inverting the polarity (+8 and +4 V, respectively). The onset voltage for FeN–S1 and FeN–S2 (−8 V) is higher than that Co–S1 and CoN–S2 (−4 V), and all films clearly recover the as-prepared *M*_S_ under inverted bias (i.e., *M*_S_ modulation is fully reversible). The measured onset voltages reflect a lower activation energy for N ion motion in cobalt nitrides than in iron nitrides, presumably due to the lower electronegativity between nitrogen and iron compared to cobalt (1.64 vs. 1.7)^66,67^ and an increased cohesive energy.^25^ FeN–S1 shows an excellent ability to completely recover not only the magnetization of as-grown film but also the squareness and slope at the coercive field (Figure S7), which could make it useful for practical applications.

CoN–S2 films were also examined via HDAAF-STEM and EELS after onset and recovery (−4 and +4 V) experiments were completed (Figure 4c–f) to establish how the film changes under threshold voltage cycling and, to lesser extent, what extent nitrogen is still present in the film, since previous HDAAF-STEM observations made after applying −50 V for 75 min showed that all nitrogen had been released to the electrolyte.^24^ After negative biasing of −4 V for 75 min, CoN–S2 undergoes a very mild change, corresponding to a slightly nanoporous structure near the electrolyte side as well as a decrease in the nitrogen content. Remarkably, under +4 V biasing, the film is nearly fully recovered with small nitrogen-deficient regions still remaining postrecovery. Additional VEPALS measurements were carried out on FeN–S1 and CoN–S2 (Figure S4) for the as-prepared films, films treated at the onset voltage (−8 and −4 V for CoN and CoN, respectively), and films treated at −50 V. It is observed, postonset treatment, that *τ*_1_ remains very similar to the as-prepared film (besides the subsurface region, where it increases) while the intensity *I*_1_ increases slightly across all films. This suggests a very mild increase in the number of local vacancy clusters under onset biasing, consistent with the limited removal of N from the film. Post −50 V treatment, a local peak in the intensity of lifetime *τ*_2_ (grain boundaries) is observed in both systems, characteristic of a migration front moving through the sample as nitrogen is removed.

**Figure 5:**
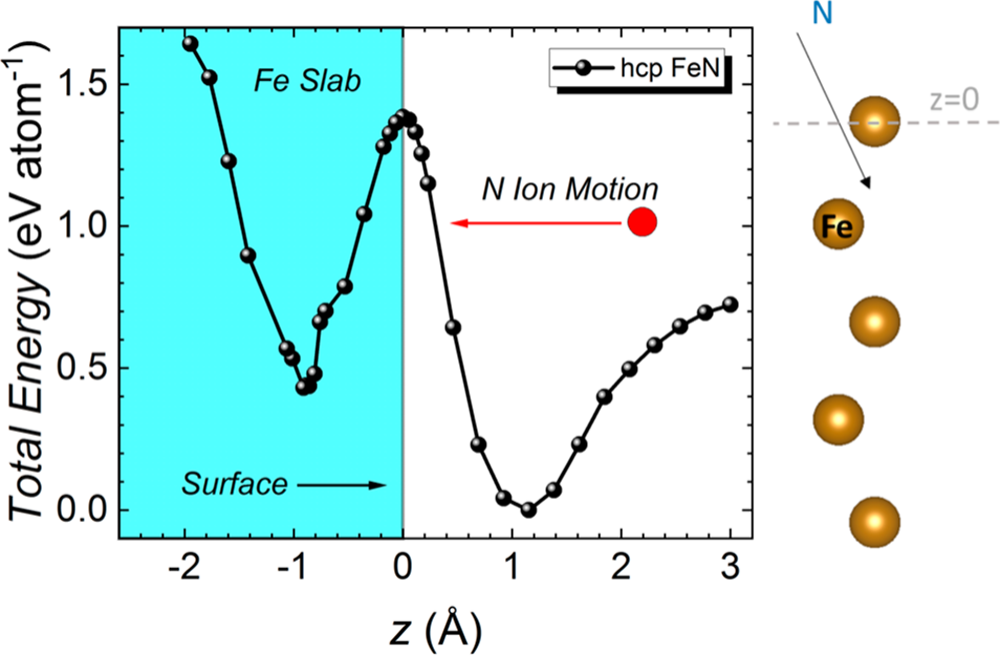
Ab initio calculations of the threshold energy FeN system. Calculated total energy per atom, normalized to the minimum energy value, as a function of the displacement between the outermost Fe surface atom and the inserted N atom for both hexagonal close-packed (hcp) structured Fe slabs. Five-monolayer-thick Fe slab schematic is shown in the right panel, where the dashed line indicates the reference *z* position, which is the outermost Fe surface monolayer.

To understand the differences in the energetics between iron and cobalt nitrides, ab initio calculation of the energy required to induce ionic motion was performed. Using the nudged elastic band method (NEB) (Methods), minimum energy pathways were calculated for the insertion of a nitrogen atom into an iron slab with hcp FeN structure. The obtained total energy per atom (normalized to the global minimum) is plotted in Figure 5 as a function of the displacement *z* of the nitrogen atom from the iron reference monolayer (top layer). Setting the outermost iron layer as *z* = 0 Å, the global energy minimum for hcp is found at *z* = 1.15 Å with another local minimum located around *z* = −0.91 Å. The critical electric field, *E*_C_, can be estimated using the electric potential per atom between minima (1.38 eV atom^–1^) required to move a nitrogen atom between minima, leading to *E*_C_ ≈ 6.6 V nm^–1^, similar to the values observed for onset in FeN–S1 and FeN–S2 (−8 V, see Figure 4a) and larger (in absolute values) than the critical electrical fields needed to induce nitrogen ion motion in CoN. These results complement recent calculations which show CoN has a lower calculated energy barrier^24^ as well as previous simulations where FeN was found to have a (slightly) higher cohesive energy than CoN.^25,68^

## Conclusion

Magneto-ionics has been demonstrated in single-layer, 85 nm thick near-stoichiometric FeN films through electrolyte gating, capable of controllably generating and removing a ferromagnetic state (ON–OFF ferromagnetism). The magneto-ionic properties of FeN are compared with CoN. FeN is found to have a greater total magnetization, a higher coercivity, and a lower rate of magnetization generation under larger bias as well as a higher onset voltage than the examined cobalt nitrides. The available open volume supporting ion transport is slightly lower for FeN as indicated by PALS. Ab initio calculations show that the formation energy of FeN requires a greater onset voltage (≈6.6 V nm^–1^) to induce magneto-ionic motion than CoN (≈5.3 V nm^–1^). The larger total magnetization and coercivity achieved upon biasing show iron-based nitrides are a viable material for magneto-ionic applications and appealing as potential materials in existing nitride semiconductor devices and memory system architectures.

## Methods

### Sample Preparation

Eighty-five nanometer thick FeN was grown by reactive sputtering on boron-doped, highly conducting [100], 500 μm thick silicon wafers, previously coated with 20 nm of titanium and 60 nm of copper. The copper was masked during deposition to serve as a working electrode.

The expanded FeN films were grown in a homemade triode sputtering system with a base pressure in the range of 10^–8^ Torr. Ultrahigh vacuum was ensured to minimize oxygen contamination. The target to substrate distances were around 10 cm, and the sputtering rate was around 1 Å s^–1^. CoN films were grown in a range of nitrogen partial pressure (100% Ar/0% N_2_, 75% Ar/25% N_2_, 50% Ar/50% N_2_) environments at a total pressure of 8 × 10^–3^ Torr.

### Magnetic Characterization

Magneto-electric measurements were performed by vibrating sample magnetometry while electrolyte gating the film in a capacitor-like configuration at room temperature. The films are mounted in a homemade electrolytic cell containing anhydrous propylene carbonate with sodium cation-solvated species (5 ppm). The Na^+^-solvated species in the electrolyte are present to react with any trace amounts of water in the propylene carbonate.^69^

The magnetic properties of the films were measured in plane while applying different voltages. This was done using a Micro Sense (LOT, Quantum Design) magnetometer with a maximum field of 20 kOe. Voltages were applied using an Agilent B2902A power supply between the working electrode and the counter electrode, as demonstrated in previous works.^12,61,69^ The magnetic signal was normalized to the volume exposed to the electrolyte during the gating process. All measured hysteresis loops were background corrected, carried out at high fields (always above the saturation field), to eliminate linear contributions (paramagnetic or diamagnetic signals).

### Structural and Compositional Measurements

The θ/2θ X-ray diffraction patterns were recorded on a Materials Research Diffractometer (MRD) from the Malvern PANalytical Co., equipped with a PIXcel^1D^ detector, using Cu Kα radiation. The XRD patterns were analyzed using Rietveld refinement to obtain lattice cell parameters and crystallite size (average size of coherently diffracting domains).^51^

High-resolution transmission electron microscopy (HRTEM), high-angle annular dark-field scanning transmission electron microscopy (HDAAF-STEM), and electron energy loss spectroscopy (EELS) were performed on a TECNAI F20 HRTEM/STEM microscope operated at 200 kV. Cross-sectional lamellae were prepared by focused ion beam, capped with a sacrificial platinum layer, and placed onto a Cu transmission electron microscopy grid.

### Transport Measurements

Both cobalt nitride and iron nitride films were deposited onto high-resistivity Si substrates. Resistivity values were acquired from 30 to 300 K, all using the van der Pauw configuration.

### Variable Energy Positron Annihilation Lifetime Spectroscopy

Variable energy positron annihilation lifetime spectroscopy (VEPALS) measurements were performed at the monoenergetic positron source (MePS) at the radiation source ELBE (Electron Linac for beams with high Brilliance and low Emittance) at Helmholtz-Zentrum Dresden-Rossendorf (Germany).^52^ A CeBr_3_ detector coupled to a digital lifetime spectrometer with homemade software employing a SPDevices ADQ14DC-2X with 14-bit vertical resolution and 2 GS s^–1^ (gigasamples per second) horizontal resolution was used. The time resolution function was estimated to be about 0.205 ns. The resolution function required for spectrum analysis uses two Gaussian functions with distinct intensities depending on the positron implantation energy, *E*_p_, and appropriate energy shifts. All spectra measured contain at least 10^7^ counts.

### Ab Initio Calculations

The first-principles calculations were based on the projector-augmented wave (PAW)^70^ method as implemented in the VASP package^71−73^ using the generalized gradient approximation.^74^ The virtual crystal approximation^75^ was used to model the variation of nitrogen per unit cell. To calculate the Fe–N formation energy, the nudged elastic band method (NEB)^76,77^ on the nitrogen pathway into a five-monolayer thick (0001) hexagonal close-packed Fe slab was used. At each step, the atomic coordinates were relaxed until the forces became smaller than 1 meV Å^–1^. A kinetic energy cutoff of 500 eV was used for the plane-wave basis set 25 × 25 × 1. *k*-point meshes were used to construct the Brillouin zone in the Fe slab in the NEB calculations.

The data used in this article are available from the corresponding authors upon request.

## Supporting Information Available

X-ray diffraction patterns of CoN films, resistivity measurements on CoN films, positron lifetime experiments for FeN and CoN films, hysteresis loops and magnetoelectric data for CoN films, and magnetoelectric recovery data for FeN films (PDF)

## Author Present Address

E.M. and J.S. had the original idea and led the investigation. J.d.R., E.M., and J.So. designed the experiments. J.Sa. and J.L.C. prepared the FeN and CoN films and performed the electrical transport measurements. F.I. and M.C. performed the ab initio calculations. A.Q. and E.M. designed the sample holder to carry out the magnetoelectric measurements. J.d.R., E.M., and J.So. carried out the magnetoelectric measurements and analyzed the data. J.d.R., A.Q., and E.M. performed the XRD characterization and analyzed the data. J.d.R., A.L., L.A., and E.M. carried out the TEM and STEM characterization and analyzed the corresponding data. M.O.L., M.B., E.H., and A.W. characterized the samples by PALS and analyzed the data. All authors discussed the results and commented on the article. The article was written by J.d.R., E.M., and J.So.
