# Supplementary material for: Magneto-Ionics in Single-Layer Transition Metal Nitrides
Source: ACS Appl Mater Interfaces. 2021 Jun 22;13(26):30826–34. doi: 10.1021/acsami.1c06138 (PMC8483439; doi:10.1021/acsami.1c06138)
Supplement: Supplementary file 1 — am1c06138_si_001.pdf [file am1c06138_si_001.pdf]

Supporting Information

Magneto-ionics in single-layer transition metal nitrides

Julius de Rojas[1], Joaquín Salguero[2], Fatima Ibrahim[3], Mairbek Chshiev[3,4], Alberto Quintana[5], Aitor Lopeandia[1,6], Maciej O. Liedke[7], Maik Butterling[7], Eric Hirschmann[7], Andreas Wagner[7], Llibertat Abad[8], José L. Costa-Krämer[2], Enric Menéndez[1] and Jordi Sort[1,9*]*

[1] Departament de Física, Universitat Autònoma de Barcelona, E-08193 Cerdanyola del Vallès, Spain

[2] IMN-Instituto de Micro y Nanotecnología (CNM-CSIC), Isaac Newton 8, PTM, 28760 Tres Cantos, Madrid, Spain

[3] Univ. Grenoble Alpes, CEA, CNRS, Spintec, 38000 Grenoble, France

[4] Institut Universitaire de France, 75231 Paris, France

[5] Department of Physics, Georgetown University, Washington, D.C. 20057, United States

[6] Catalan Institute of Nanoscience and Nanotechnology (ICN2), Campus UAB, Bellaterra, Barcelona, 08193, Spain

[7] Institute of Radiation Physics, Helmholtz-Zentrum Dresden – Rossendorf, Dresden 01328, Germany

[8] Institut de Microelectrònica de Barcelona, IMB-CNM (CSIC), Campus UAB, E-08193 Bellaterra, Spain

[9] Institució Catalana de Recerca i Estudis Avançats (ICREA), Pg. Lluís Companys 23, E-08010 Barcelona, Spain

Corresponding Authors

*E-mail: enric.menendez@uab.cat (Enric Menéndez)

*E-mail: jordi.sort@uab.cat (Jordi Sort)

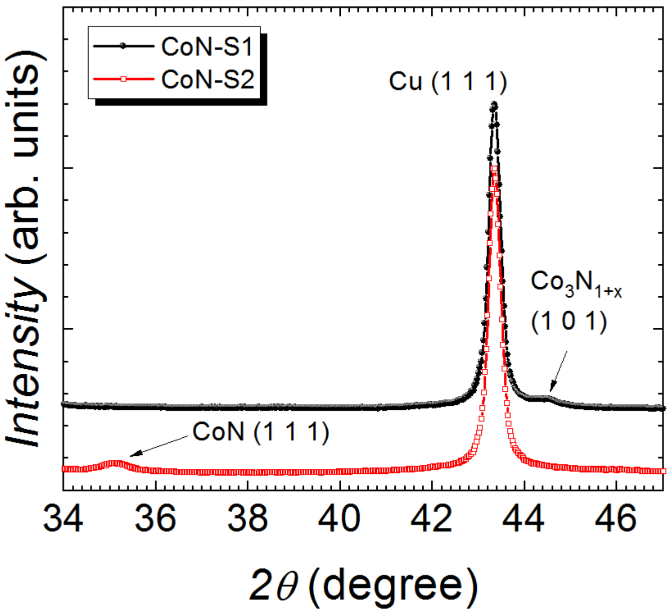

Figure S1. Structural characterization via θ/2θ X-ray diffraction (XRD of as-prepared CoN-S1 and CoN-S2 films).

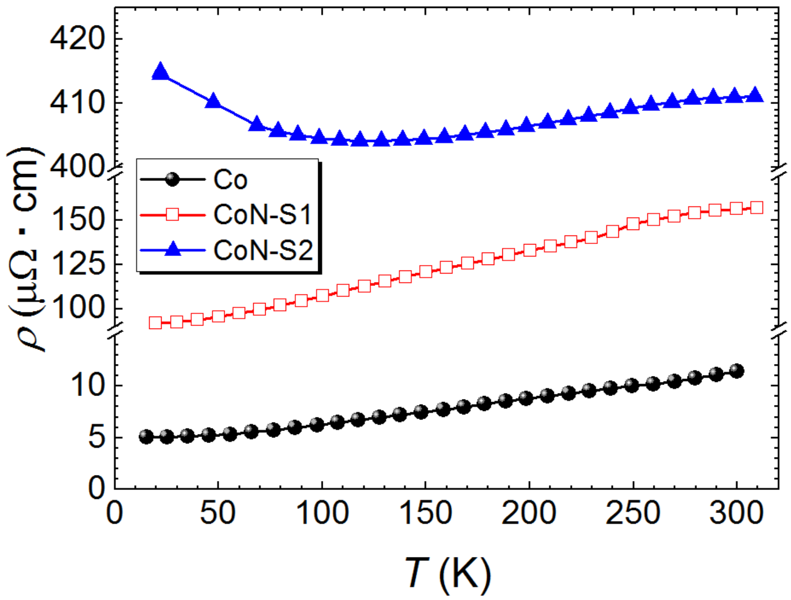

Figure S2. Measured resistivities, ρ, as a function of temperature, T, for pure Co, CoN-S1 and CoN-S2 films.

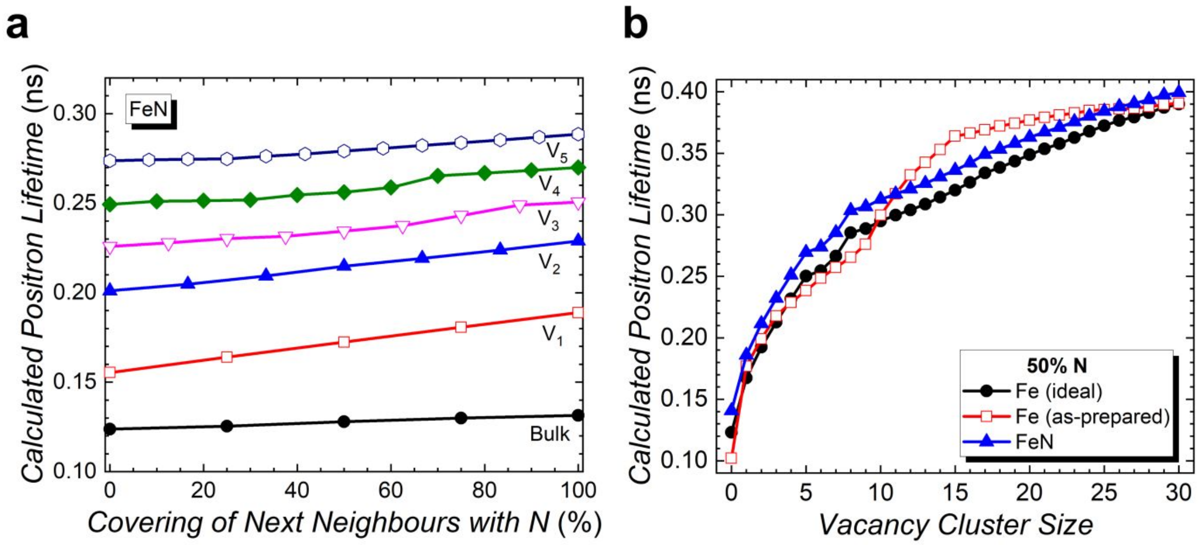

Figure S3. (a) Positron lifetime for different annihilation states depending on the N concentration for as-prepared FeN. V_i, i = 1-5 represents a number of Fe vacancies within a cluster, which are decorated with an increasing number of nitrogen atoms between zero N nearest neighbors (0%) and fully surrounded (100%). (b) Calculated positron lifetimes for ideal, as-prepared, and nitrided systems (FeN), depending on the number of clustered vacancies. Calculations performed using ATSUP method.

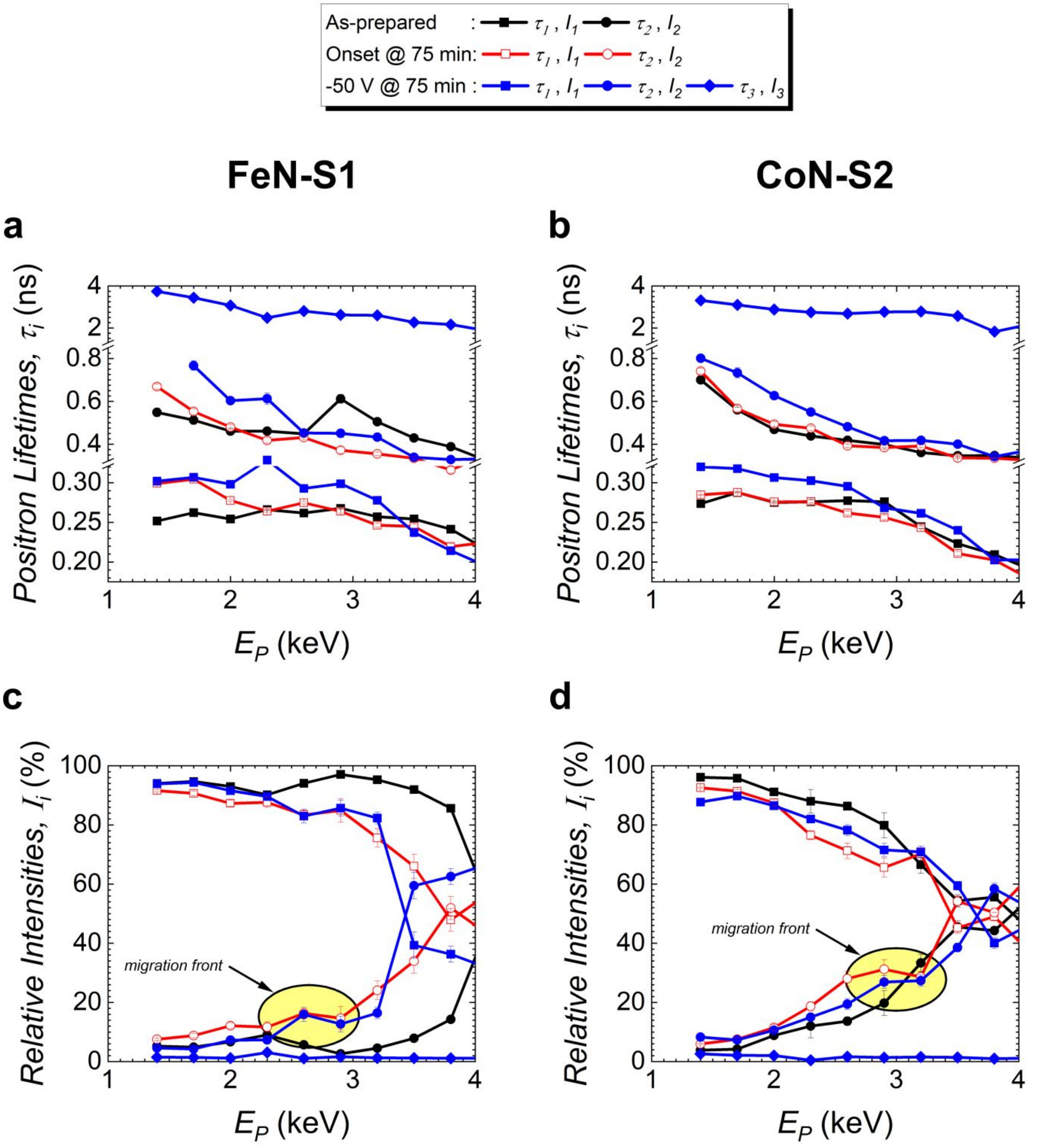

Figure S4. Defect characterization by variable energy positron annihilation lifetime spectroscopy (VEPALS). (a-b) Positron lifetime components $\tau_{i=1\text{-}3}$ and **(c-d)** relative intensities $I_{i=1\text{-}3}$ as a function of positron implantation energy E_P for FeN-S1 and CoN-S2, respectively. As-prepared, onset, and -50 V

biased plots are shown. Migration fronts noted with yellow circles for FeN and CoN at 2.5 keV and 3.5 keV, respectively.

For PALS data acquisition a Teledyne SPDevices ADQ14 DC 2X MTCA digitizer with 2 GS s^{-1}, 14 bit and pulse detection performed on an integrated FPGA was utilized. In order to acquire the stop time and, hence, the positron annihilation event (positron lifetime), a simplified interpolation method based on Vandermonde interpolation, in combination with Householder root-finding, was utilized. Time determination using the Householder method made it possible to replace many complex calculations without losing the required precision. Parallel programming maximized throughput rates by sequencing various dependencies. As a result, the digital data acquisition was comparable (or better) to the analog measurement electronics [for details see http://dx.doi.org/10.25673/35683 (German only); a technical manuscript written in English is in preparation]. A precise estimation of start/stop times is crucial for positron lifetime evaluation; however, it does not introduce additional errors beyond those already included in the spectrum resolution function, which is subsequently fitted using the PALSfit code. The error bars in all PALS figures represent standard deviations of the fitting parameters (for details see http://palsfit.dk/ and https://backend.orbit.dtu.dk/ws/portalfiles/portal/130663792/palsfit3_final.pdf). They are obviously smaller than visible, but relatively small scattering of data points for different positron implantation energies E_p. A systematic error which is not included here is expected to be larger than standard deviations; however, there is no simple way to calculate it. The systematic error is affected by numerous factors, e.g., sample size (when compared to the positron beam diameter of 5-6 mm), slight variations of the beam diameter per E_p (normally below several percent), bunching efficiency per E_p, or varied signal intensity of reflected positrons per E_p (normally

below 0.5%). Nevertheless, in all presented PALS results even a combination of systematic and standard deviation errors will be smaller than observed positron lifetime changes between samples. Moreover, scattering of the points is not necessarily purely due to systematic errors, and usually reflects local variations of the defect microstructure at a certain depth. It is however convoluted with positron diffusion length and local defect charge states. Since the beam contains a large enough number of positrons to localize most of available defects within a certain depth, the obtained information about defect size is, to some extent, averaged out. Nevertheless, exponential decomposition of PALS spectra allows the separation of these different contributions and the estimation of these defect types and sizes. The PALS sensitivity is not strictly local, as in the case of imaging techniques, but more global, volumetric, which in our opinion carries a significant advantage.

The effect of biasing on both the structure of both films was also measured, at the onset voltage (-8 V and -4 V for FeN-S1 and CoN-S2, respectively) and -50 V. In FeN-S1 under -8V, increases in τ_1 and τ_2 (and the appearance of τ_3) are observed within the first 25 nm of the film, indicating existing open volumes increase in size near the surface. In addition, these changes coincide with a relative decrease in I_1 and increase in I_2, scaling with increasing depth. These changes suggest that small vacancies develop into mixed boundary vacancies deeper into the film, while as the top vacancies increase in size. This confirms the general change in structure post-biasing seen with HDAAF-STEM (Figure 3), where a more “nanoporous” phase is seen

near the electrolyte-side. CoN-S2 shows both τ_1 and τ_2 both increasing relative to the as-prepared state, as well as the appearance of τ_3 (voids) is observed, although quite residual. At -4V as well as -50 V, an increase in relative intensity I_1 and I_2 maxima are observed, coinciding with the locations of the "diffusion fronts" seen in TEM images (Figure 3g), near 3 keV. At -50 V, FeN-S1 shows a more dramatic increase in τ_1 and τ_2 (in particular τ_2, which shows tremendous change within the first 30 nm of the surface), as well as feature associated with a migration front near 2.5 keV. As τ_2 approaches the onset value deeper in the surface, the intensities I_1 and I_2 show dramatic decreases and increases, respectively, evidencing that closer to the electrode vacancies transform in character from small clusters to mixed grain boundary vacancies. This is consistent with the behavior of the film treated at -8 V, where the dominant behavior near the surface is an increase in open volume (which increases with voltage), and near the electrode a transformation of vacancy character (cluster to grain boundary), which extends further in the film with increased voltage. This could explain the remarkable way in which iron nitride is able to completely recover its magnetic state (Figure S7). In contrast to FeN-S1, the migration front of CoN-S2 moves deeper into the film, as well as an overall increase in τ_1 and τ_2, consistent with a complete denitriding process.

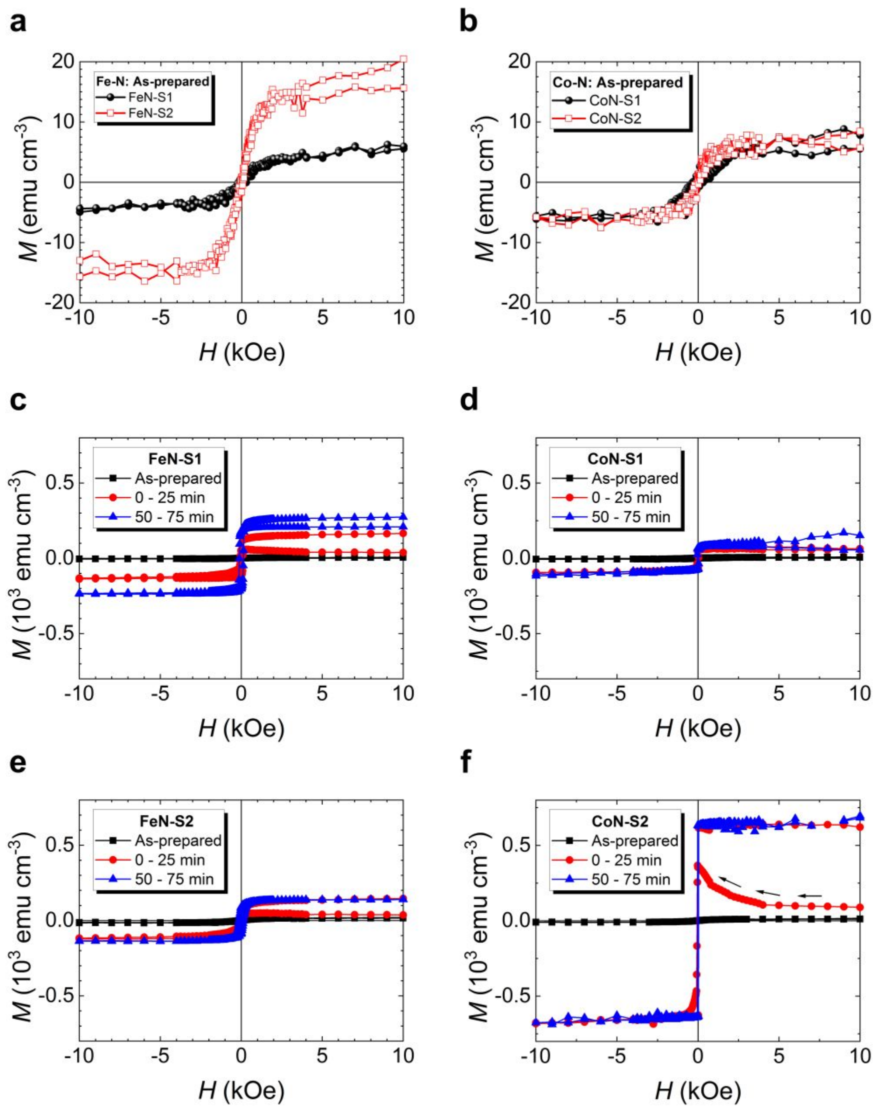

Figure S5. (a-b), as-prepared hysteresis loops for iron and cobalt nitride films respectively. **(c,e)** are magnetic hysteresis loops measured consecutively under -50 V gating for FeN-S1 and FeN-S2, respectively. **(d,f),** magnetic hysteresis loops measured consecutively under -50 V gating for CoN-S1 and CoN-S2, respectively. Loops were measured in the in-plane configuration using a vibrating sample magnetometer.

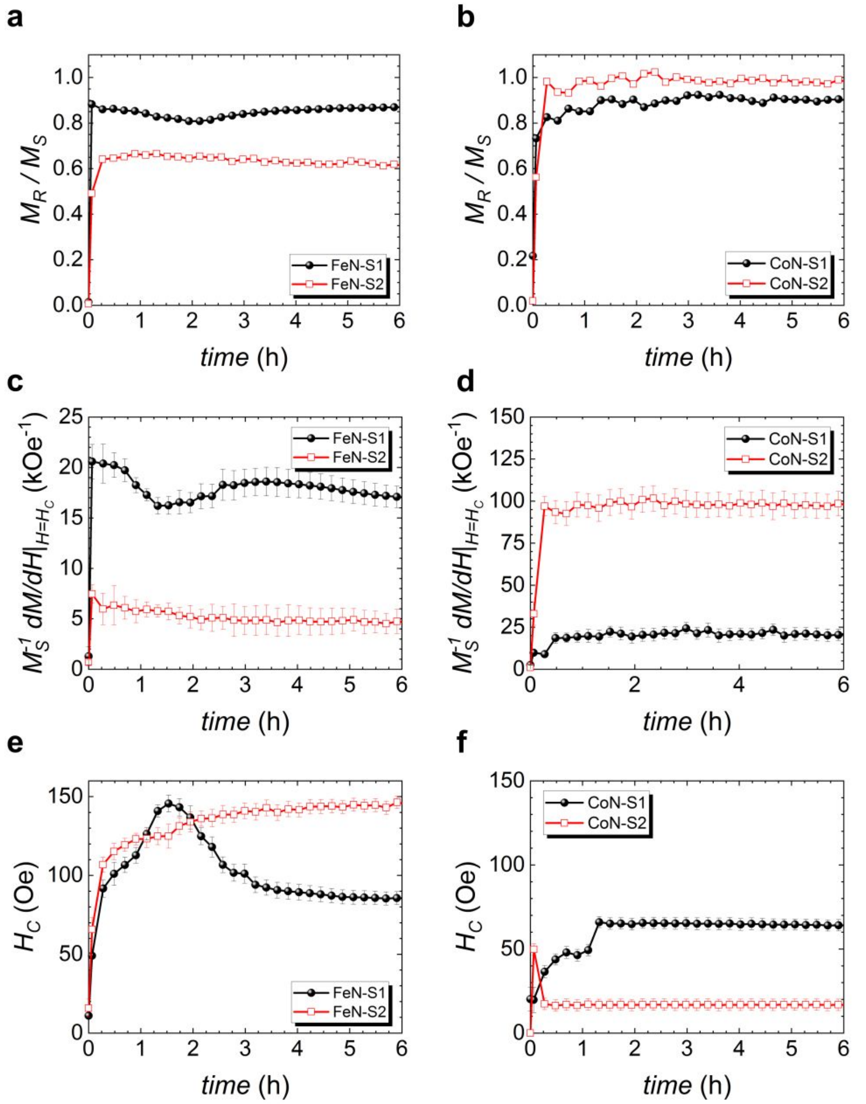

Figure S6. Magnetoelectric characterization of iron and cobalt nitride films. (a-b), time evolutions of squareness (M_R/M_S). **(c-d)**, time evolutions of the slope of the *M-H* curve, evaluated at the coercive field, normalized to the saturation magnetization of each loop ($M_S^{-1} dM/dH\ [H=H_C]$). **(e-f)**, time evolutions of coercive field (H_C). Values measured consecutively under -50 V gating.

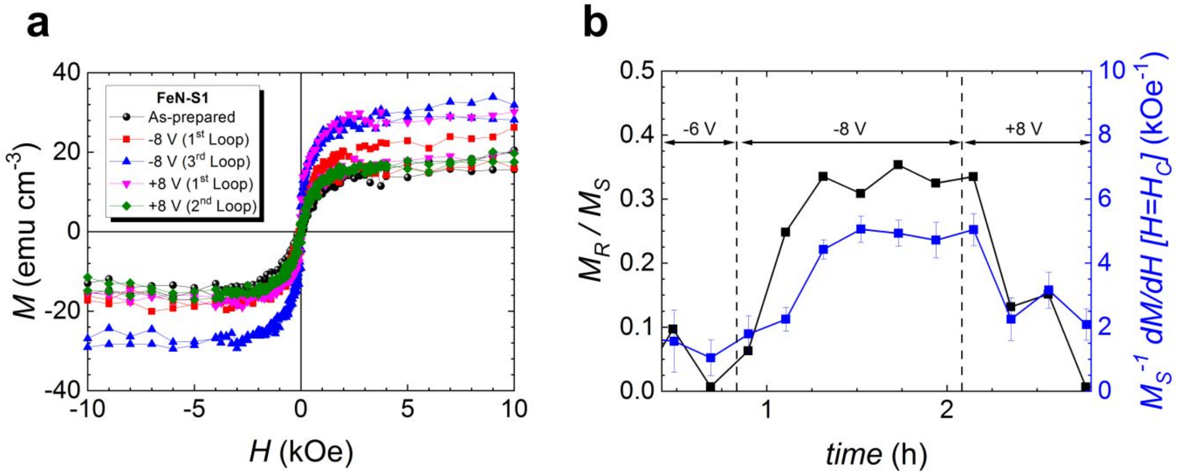

Figure S7. (a) Magnetic hysteresis loops measured consecutively under onset (-8 V) and recovery (+8 V) gating for the FeN-S1 film. **(b)** Squareness and slope at coercive field as measured during the onset/recovery gating.
